# Seasonal migrations of North Atlantic minke whales: novel insights from large-scale passive acoustic monitoring networks

**DOI:** 10.1186/s40462-014-0024-3

**Published:** 2014-11-18

**Authors:** Denise Risch, Manuel Castellote, Christopher W Clark, Genevieve E Davis, Peter J Dugan, Lynne EW Hodge, Anurag Kumar, Klaus Lucke, David K Mellinger, Sharon L Nieukirk, Cristian Marian Popescu, Christian Ramp, Andrew J Read, Aaron N Rice, Monica A Silva, Ursula Siebert, Kathleen M Stafford, Hans Verdaat, Sofie M Van Parijs

**Affiliations:** Under Contract with Northeast Fisheries Science Center, National Marine Fisheries Service, NOAA, Woods Hole, MA USA; Scottish Association for Marine Science (SAMS), Scottish Marine Institute, Oban, Argyll, Scotland UK; National Marine Mammal Laboratory, Alaska Fisheries Science Center, National Marine Fisheries Service, NOAA, Seattle, WA USA; Bioacoustics Research Program, Laboratory of Ornithology, Cornell University, Ithaca, NY USA; Duke University Marine Laboratory, Beaufort, NC USA; Naval Facilities Engineering Command Atlantic, Norfolk, VA USA; IMARES Wageningen UR, Ecosystem Department, Den Burg, Texel Netherlands; Centre for Marine Science & Technology, Curtin University, Perth, WA Australia; NOAA Pacific Marine Environmental Laboratory, Newport, OR USA; Cooperative Institute for Marine Resources Studies, Oregon State University, Newport, OR USA; Mingan Island Cetacean Study, Longue-Pointe-de-Mingan, QC Canada; MARE-Marine and Environmental Sciences Centre and IMAR-Institute of Marine Research, University of the Azores, Horta, Portugal; Biology Department, Woods Hole Oceanographic Institution, Woods Hole, MA USA; Institute for Terrestrial and Aquatic Wildlife Research, University of Veterinary Medicine Hannover, Foundation, Büsum Germany; Applied Physics Laboratory, University of Washington, Seattle, WA USA; Northeast Fisheries Science Center, National Marine Fisheries Service, NOAA, Woods Hole, MA USA

**Keywords:** Passive acoustic monitoring (PAM), Minke whales, *Balaenoptera acutorostrata*, Migration, Pulse trains, Seasonality, Geographic variation

## Abstract

**Background:**

Little is known about migration patterns and seasonal distribution away from coastal summer feeding habitats of many pelagic baleen whales. Recently, large-scale passive acoustic monitoring networks have become available to explore migration patterns and identify critical habitats of these species. North Atlantic minke whales (*Balaenoptera acutorostrata*) perform seasonal migrations between high latitude summer feeding and low latitude winter breeding grounds. While the distribution and abundance of the species has been studied across their summer range, data on migration and winter habitat are virtually missing. Acoustic recordings, from 16 different sites from across the North Atlantic, were analyzed to examine the seasonal and geographic variation in minke whale pulse train occurrence, infer information about migration routes and timing, and to identify possible winter habitats.

**Results:**

Acoustic detections show that minke whales leave their winter grounds south of 30° N from March through early April. On their southward migration in autumn, minke whales leave waters north of 40° N from mid-October through early November. In the western North Atlantic spring migrants appear to track the warmer waters of the Gulf Stream along the continental shelf, while whales travel farther offshore in autumn. Abundant detections were found off the southeastern US and the Caribbean during winter. Minke whale pulse trains showed evidence of geographic variation, with longer pulse trains recorded south of 40° N. Very few pulse trains were recorded during summer in any of the datasets.

**Conclusion:**

This study highlights the feasibility of using acoustic monitoring networks to explore migration patterns of pelagic marine mammals. Results confirm the presence of minke whales off the southeastern US and the Caribbean during winter months. The absence of pulse train detections during summer suggests either that minke whales switch their vocal behaviour at this time of year, are absent from available recording sites or that variation in signal structure influenced automated detection. Alternatively, if pulse trains are produced in a reproductive context by males, these data may indicate their absence from the selected recording sites. Evidence of geographic variation in pulse train duration suggests different behavioural functions or use of these calls at different latitudes.

**Electronic supplementary material:**

The online version of this article (doi:10.1186/s40462-014-0024-3) contains supplementary material, which is available to authorized users.

## Background

Animal migration is a common phenomenon and has evolved at multiple times and in a variety of species [1]. Typically, migration develops as an adaptation to take advantage of seasonal peaks in resource abundance, escape inter- and intra-specific competition, or avoid predators and parasites [2]. Most baleen whale species perform to-and-fro migrations [3] between productive high latitude summer feeding and low latitude winter breeding grounds [4,5] and have been shown to cover very large distances, including the longest documented migration distance by any mammal [6]. The driving forces for these long-range migrations to often unproductive breeding grounds are still debated and a number of explanations have been suggested, including increased calf survival and avoidance of killer whale (*Orcinus orca*) predation [7]. However, there is also increasing evidence that partial (a fraction of the population stays on the feeding grounds) or differential (differences in migratory behaviour between different age classes or sexes) migration [3,8] might be more the norm than the exception in baleen whales. For example, several long-term passive acoustic monitoring (PAM) studies show the extended year-round presence of baleen whales on higher-latitude feeding grounds [9-12]. Nonetheless, at least parts of most populations of baleen whales seasonally migrate between summer feeding and winter breeding grounds [13-17].

Due to the high mobility of individuals, short surface times and the dependence on daylight and favorable weather conditions, it is generally difficult to visually survey for marine mammals. These limitations are intensified during migration, when their locations and movements are generally less predictable. Thus, baleen whale migration routes in the North Atlantic Ocean are still poorly understood for most species. In addition, while summer and winter destinations are fairly well described for the more coastally distributed species such as humpback (*Megaptera novaeangliae*) and right whales (*Eubalaena glacialis*) [18,19], little is known about the winter distribution of most other baleen whale species in the North Atlantic. For blue (*Balaenoptera musculus*) and fin whales (*Balaenoptera physalus*) there are some passive acoustic data indicating low latitude winter distributions [20,21], while more recent recordings also suggest year-round presence in higher latitudes [9,11]. Apart from these observations most knowledge on migration routes still originates from historical whaling records [22,23].

Such lack of data is not limited to baleen whales, but extends to other long-distance migrants that spend much of their lives in open ocean regions, such as sea turtles and pelagic seabirds [24,25]. Given current ocean-scale impacts of climate change and an increase in offshore, anthropogenic activities [26-28], a better understanding of migration timing and the location and extent of migration corridors of highly mobile marine mammals and other top predators is crucial for effective marine conservation efforts, which are currently concentrated in coastal habitats [24,29,30]. New methods such as statistical modeling, electronic tracking, as well as PAM are emerging as promising tools to gather such fundamental information on marine mammal movement and seasonal habitats [30-33].

Although North Atlantic minke whales (*Balaenoptera acutorostrata*) are well studied on their summer foraging grounds [34-38], large knowledge gaps exist concerning their distribution and abundance for much of the rest of the year. As far as it is known, their range extends from Baffin Bay to the Caribbean in the western North Atlantic and from the Barents Sea to the African continental shelf in the eastern North Atlantic [39,40]. Similar to the life cycle of other baleen whales, there is evidence of large-scale seasonal migrations between summer feeding in higher latitudes and winter breeding grounds in lower latitudes [39,41], but winter habitats have not been identified for this species. North Atlantic minke whales are currently listed as a species of least concern under the IUCN Red List [40], but are still commercially hunted in significant numbers in the North Atlantic. Based on limited data from feeding grounds, the International Whaling Commission (IWC) partitions North Atlantic minke whales into four discrete management areas: the Canadian East coast stock, the West Greenland stock, the Central stock (Iceland) and the Northeastern stock (Norway) [42]. However, there is increasing evidence for the possible existence of two breeding populations in the North Atlantic, but lack of genetic structure suggests extensive movements across and mixed assemblages at summer feeding grounds [43-45]. To confirm these data, it is important to establish the location of and obtain genetic samples from minke whale winter breeding grounds. This could have important impacts for the conservation of the species, because potential differences in genetic variability between breeding populations, for which the proportional representation in summer feeding and hunting grounds is unknown, may lead to overexploitation of small populations [43].

**Figure 1:**
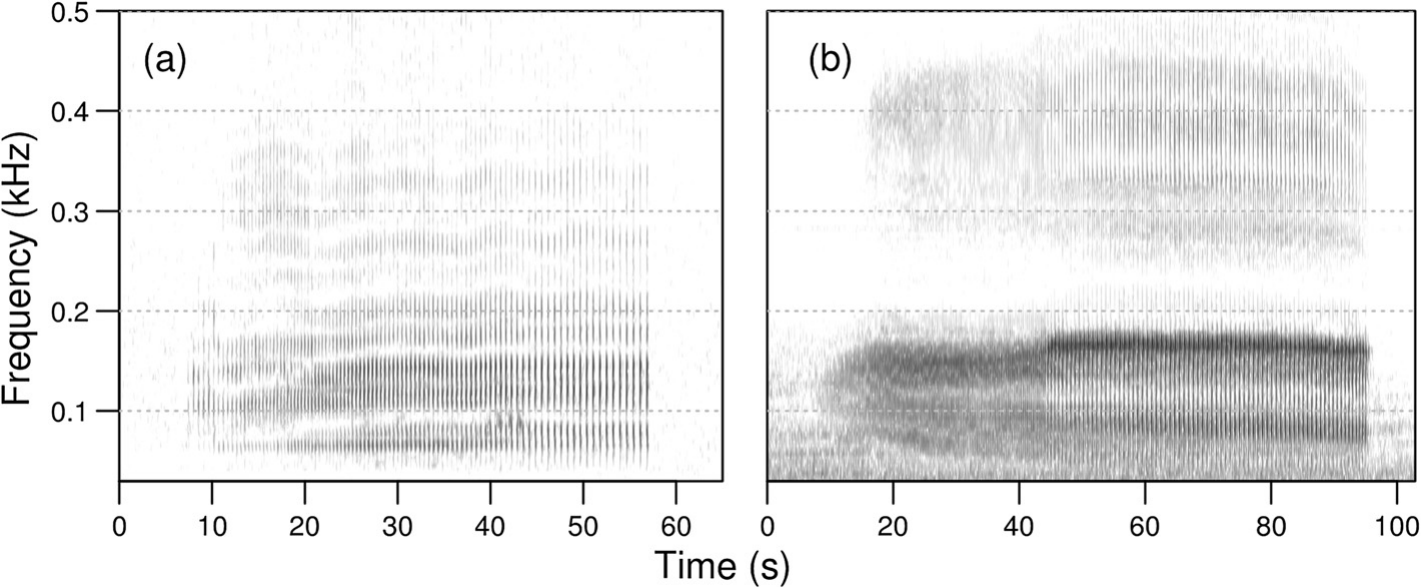
**Spectrograms for slow-down pulse train (sd3) (a) from Stellwagen Bank (site 4) and (b) from Jacksonville 2 (site 8) (see Figure** 2 **for overview map).** Spectrogram parameters: fast Fourier transform (FFT): size = 512 points, overlap = 75%, sample rate = 2000 Hz, resolution = 3.9 Hz and 64 ms. Y-axis starts at 0.03 kHz to remove low-frequency noise. Spectrograms made with Seewave [59].

A general lack of winter sightings in coastal waters of the North Atlantic, reports of a few scattered sightings [39,46] and recent aerial surveys [47] observing minke whales east of the North American continental shelf-break, suggest an offshore distribution at that time of year. Recent satellite tracking data from Iceland show that individuals that feed in Icelandic waters during summer migrate south in the middle of the North Atlantic [48], corroborating passive acoustic detections at the Mid-Atlantic ridge [49] and offshore array data from the Integrated Undersea Sound Surveillance System (IUSS-SOSUS) that showed higher counts of individual singers in lower latitudes during winter [50]. Compared to the acoustic signals of other baleen whale species, until recently, minke whale sounds in the North Atlantic have not been studied extensively. While [51] described series of clicks in the 5–6 kHz range and [52] attributed low-frequency downsweeps (118–80 Hz) to the species, the best described sounds associated with North Atlantic minke whales are low-frequency pulse trains with variable interpulse interval (IPI) structure and peak frequencies from 55–150 Hz (Figure 1) [50,53-55]. A recent long-term study of these pulse trains at Stellwagen Bank, USA demonstrated the feasibility of PAM to explore seasonal, diel and spatial occurrence patterns of this species [55]. With its obvious advantages in sampling remote areas over extended time periods regardless of weather conditions [31,56,57], PAM provides an effective tool for identifying the location and expanse of migratory corridors, especially when acoustic recorders are deployed in large spatial networks. In addition, PAM data can provide valuable information about the timing of migration periods and thus complement visual observations or satellite tracking data. Furthermore, in remote offshore areas PAM may be useful in delineating seasonally important habitats that are difficult to survey using other methods [58]. The main aims of this study were to explore the geographic and seasonal variation in minke whale pulse train occurrence across multiple sites in the North Atlantic Ocean in order to better understand minke whale seasonal and spatial movement patterns. Data from locations ranging from Nova Scotia to the Caribbean in the western North Atlantic were analyzed in detail, in order to describe migration timing and a possible migration corridor along the North American continental shelf. Data from Florida and the Caribbean were used to explore the suggested winter distribution of this species in waters off the southeastern US. Finally, geographic variation in minke whale pulse train structure was examined in order to investigate possible variation in minke whale acoustic behavior across regions.

## Results

### Ambient noise analysis and estimated maximum detection ranges

**Figure 2:**
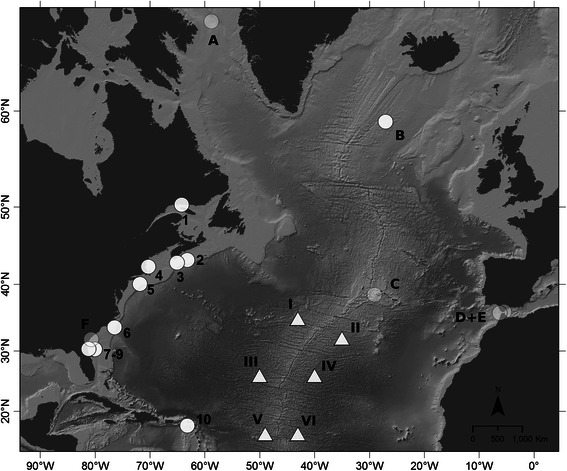
**Overview map of all North Atlantic recording sites available for this project.** Circles indicate recording sites analyzed in this study. Triangles show recording sites at the Mid-Atlantic ridge analyzed by [49] for reference. Transparent symbols show sites without detections, while white filled symbols indicate the detection of minke whale pulse trains at this site. With the exception of site 9, numbered sites 1–10 had more than 5 detections and results are shown in Figure 4. Sites A and C-F, had no detections. Site A = Davis Strait, B = SW Iceland, C = Azores, D = Cape Espartel East, E = Strait of Gibraltar West, F = Savannah. Site 1 = Gulf of St. Lawrence, 2 = Roseway Basin, 3 = Emerald Basin, 4 = Stellwagen Bank, 5 = New York, 6 = Onslow Bay, 7–9 = Jacksonville 1–3, 10 = Saba Bank. Site I-VI = NW, NE, CW, CE, SW, SE hydrophones deployed at the Mid-Atlantic ridge. Map made with data downloaded from Natural Earth. Free vector and raster map data @ naturalearthdata.com. Map projection: Mercator.

**Figure 3:**
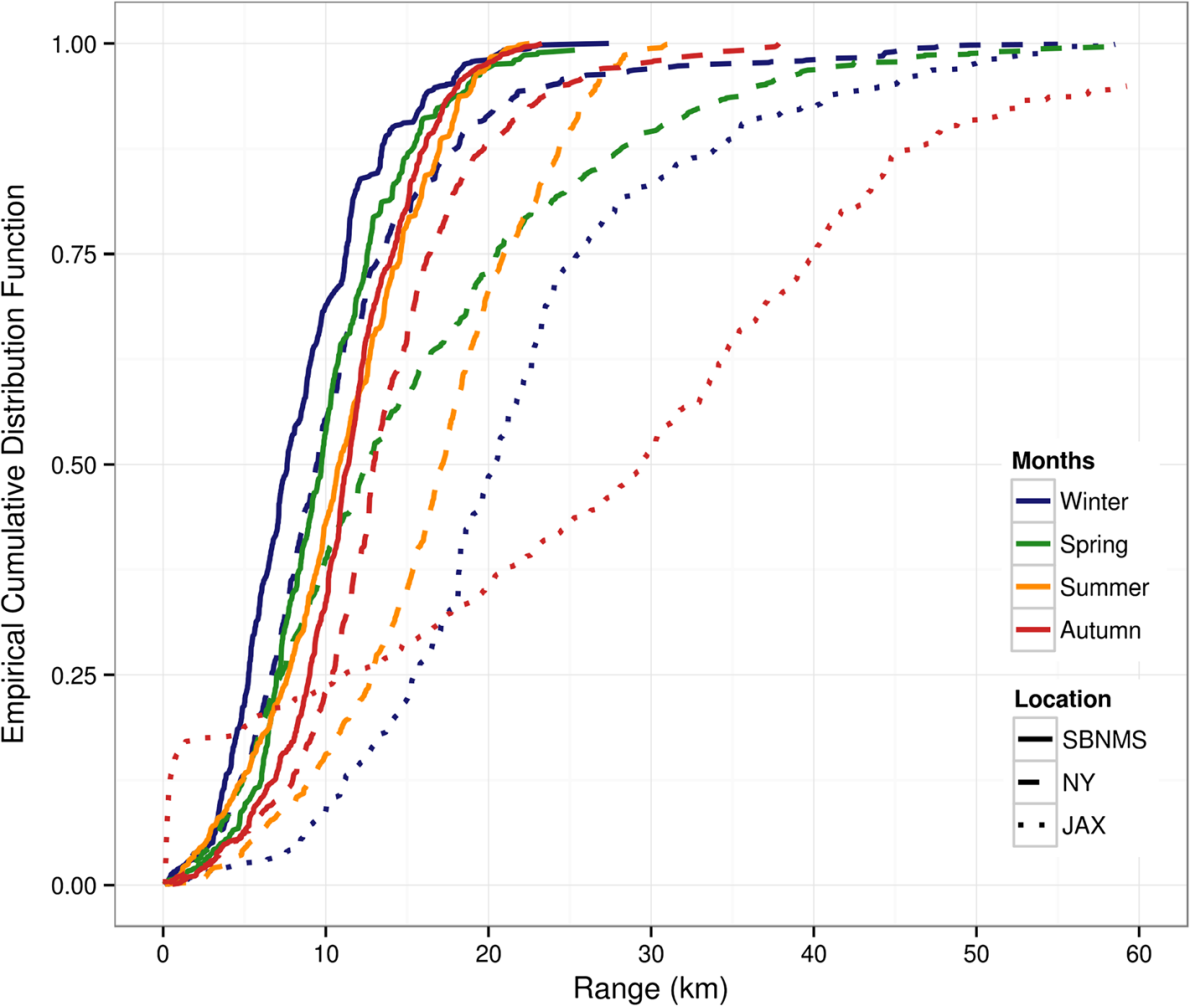
**Cumulative distribution of estimated detection ranges at sites 4, 5 and 8 (Stellwagen Bank (SBNMS), New York (NY), Jacksonville 2 (JAX); see Figure** 2 **for overview map).** Estimates are derived from ambient noise analyses of a subset of data (see Additional file 2: Table S1) and propagation modeling using the BELLHOP model, as implemented in ESME at the different locations and for all four seasons [60].

Ambient noise levels within the 89.1–355 Hz frequency bands varied spatially and temporally (Table 1). Overall, lowest median noise levels (93.09 dB re 1 μPa) were measured for Jacksonville (site 8, Figure 2) and differed from the highest median levels (105.08 dB re 1 μPa) measured at Stellwagen Bank (site 4, Figure 2) by 12 dB. Ambient noise levels for recording sites at Stellwagen Bank and New York (site 5, Figure 2) were similar in all seasons. For both sites noise levels were higher during winter and spring, as compared to data from summer and autumn months. Based on these ambient noise level measurements, estimated detection ranges for minke whale pulse trains were compared between sites and seasons. While median detection ranges for sources at Stellwagen Bank and New York are between 7.6 and 17.2 km, median detection ranges for the Jacksonville site are about 10 km greater, ranging between 20.4 and 29.4 km (Table 1, Figure 3).

**Table 1:** **Median, 25th and 75th percentile ambient noise levels (NL) measured as RMS pressure over ΔT = 600 s and over one-third octave bands 20–25 (89.1–355 Hz) for locations at Stellwagen Bank (site 4; SBNMS), New York (site 5; NY) and Jacksonville 2 (site 8, JAX) (see Figure** 2 **for overview map), across four seasons; and estimated maximum communication ranges based on a BELLHOP propagation model and the ambient noise levels above**

	**Location (site)**	**Winter**	**Spring**	**Summer**	**Autumn**
**NL (RMS)**	SBNMS (4)	105.08 (103.08, 107.31)	102.96 (100.50, 104.81)	99.74 (97.80, 103.42)	99.38 (97.01, 101.53)
**(dB re 1 μPa [89.1–355 Hz])**	NY (5)	104.10 (102.99, 106.08)	103.19 (100.55, 105.52)	96.07 (94.13, 98.83)	100.10 (98.30, 102.04)
	JAX (8)	93.12 (90.00, 95.28)	–	–	93.09 (90.58, 99.89)
**Range (km)**	SBNMS (4)	7.62 (5.26, 11.25)	9.74 (7.01, 12.56)	10.81 (7.68, 14.58)	11.40 (9.02, 14.12)
	NY (5)	9.45 (6.69, 13.61)	12.49 (7.30, 20.55)	17.18 (12.86, 20.95)	12.95 (10.43, 16.42)
	JAX (8)	20.40 (15.55, 25.55)	--	--	29.47 (11.18, 40.16)

### Seasonal and spatial variation of pulse train occurrence

A total of 3858 days of recordings were analyzed and 9411 minke whale pulse trains were detected during this analysis. The number of detections varied by geographic location and season. No pulse trains were detected in datasets from Davis Strait, the Azores, the Strait of Gibraltar or Savannah (sites A, C-F; Figure 2).

**Figure 4:**
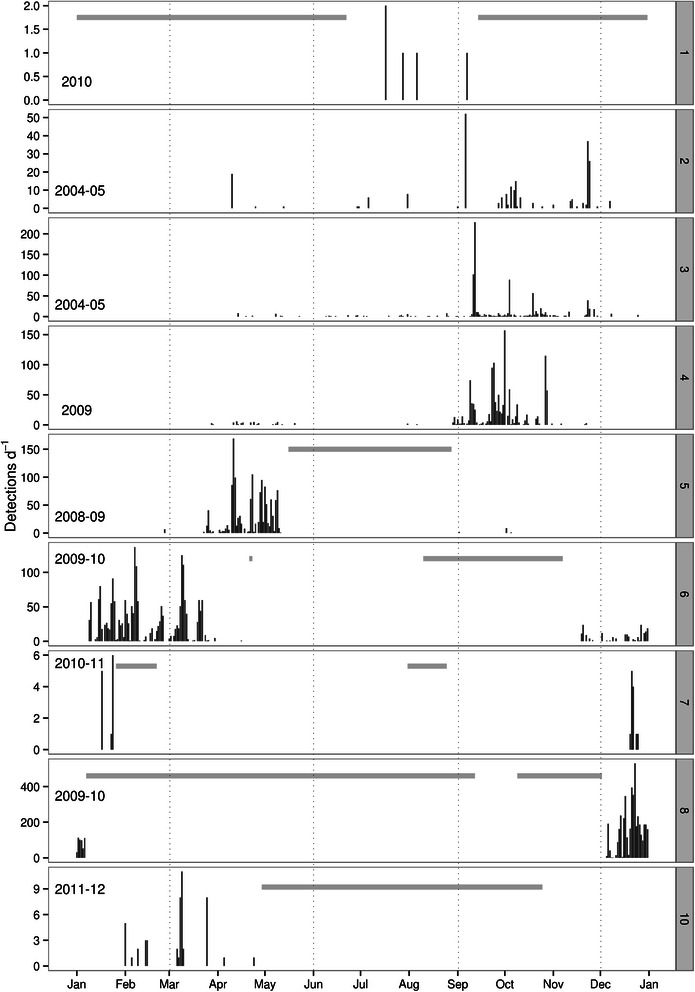
**Minke whale pulse train detections per day for all sites with more than 5 detections.** Data are presented for one fictional, continuous year to show seasonality by site. True recording years are indicated in lower left corner on each panel. Missing data indicated by grey horizontal bars. Panel numbers correspond to numbered sites in overview map (see Figure 2). Note the different y-axes scales for each panel.

While most detections were made along the US east coast, where most the recording effort was located, one pulse train was detected on the recorder deployed off Southwest Iceland (site B; Figure 2) on October 21st 2007, and 48 detections were made at the Saba Island site in the Caribbean during winter and spring (February to April; site 10; Figure 2). The seasonal distributions of minke whale pulse trains for sites with at least five detections (sites 1–8 & 10; Figure 2) are summarized in Figure 4. During the 2.5 months of summer (June to August) recordings in the Gulf of St. Lawrence (site 1; Figure 2) only five pulse trains were detected. Recording sites in Nova Scotia (sites 2 & 3; Figure 2) and Stellwagen Bank (site 4; Figure 2) all showed a peak in detections in autumn and early winter (early September to December). These sites had no detections in winter (late December to March), and only a few detections in spring and summer (April to August). In contrast, at the New York recording site (site 5; Figure 2) a peak of detections occurred in spring (mid-March to mid-May). While there was no summer data available for this site, only a few detections occurred here in autumn and none in winter. In Onslow Bay (site 6; Figure 2) most of the detections occurred during winter and spring (December to early April). No pulse trains were recorded from late April to early August and there was a gap in recording effort from late August to November. All recording sites in Jacksonville (sites 7–9; Figure 2) had detections during winter. While recordings for site 8 were only available from September to October and December to January, site 7 had gaps in recording in February and August (Figure 4).

**Figure 5:**
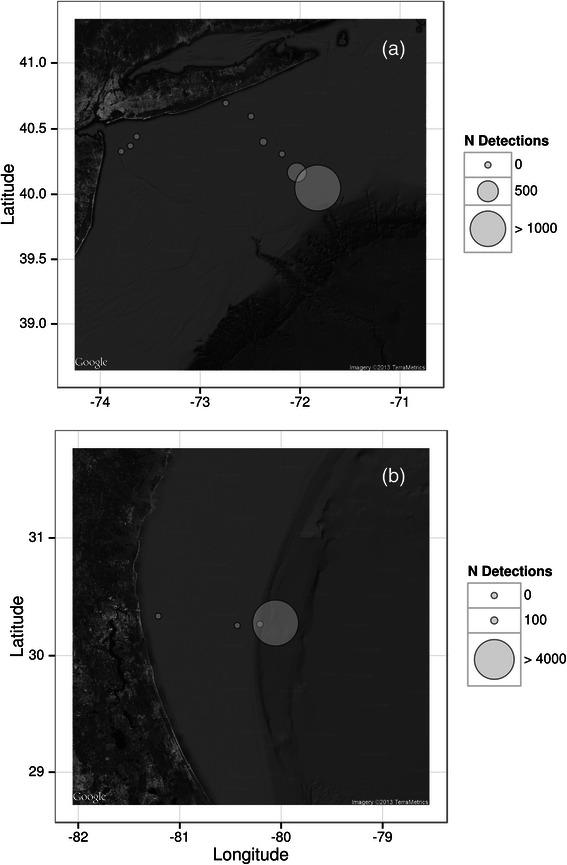
**Maps illustrating the spatial distribution of all minke whale pulse trains detected at recorders located at sites (a) New York (site 5) and (b) Jacksonville (sites 7–9) (see Figure** 2 **for overview map).**

Exploring the spatial distribution of pulse train occurrence at the New York and Jacksonville recording sites (sites 5, 7–9; Figure 2) revealed that at both recording locations the overwhelming majority of pulse trains were detected on the easternmost recording sites, which were located farthest from the coast and closest to the edge of the shelf break (Figure 5).

### Geographic variation

**Figure 6:**
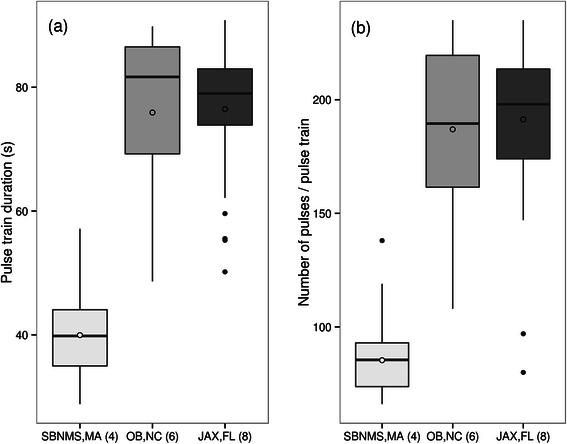
**Box-and-Whisker plot of (a) pulse train duration (s) and (b) number of pulses per pulse train at three different geographic locations: Stellwagen Bank (site 4), Onslow Bay (site 6), Jacksonville 2 (site 8) (see Figure** 2 **. for overview map).** Lower and upper bounds of boxes represent lower and upper quartiles, respectively. Solid lines represent medians and non-filled circles are means. Whiskers represent furthest data points within 1.5 × interquartile range (IQR). Filled dots are outliers.

Duration measurements were positively correlated with SNR for data from Jacksonville (R^2^ = 0.2094, p = 0.006) but not for Onslow Bay (R^2^ = 0.1274, p = 0.073) or Stellwagen Bank (R^2^ = 0.0164, p = 0.298) (Additional file 1: Figure S1). The comparison of duration and number of pulses for pulse train type sd3 (Figure 1) revealed significant differences between pulse trains recorded at Stellwagen Bank as compared to both Onslow Bay and Jacksonville (sites 4, 6, 8; Figure 6). The null hypothesis that the duration and number of pulses is equal across the three different sites was rejected (Kruskal-Wallis test: (a) pulse duration: *Χ*^*2*^ = 93.3, df = 2, p <0.001; (b) pulse number: *Χ*^*2*^ = 90.1, df = 2, p <0.001). Comparisons between Stellwagen Bank and Onslow Bay, and Stellwagen Bank and Jacksonville, showed significant differences in both pulse duration, as well as the number of pulses per pulse train (p <0.001). No significant differences were found between pulse trains recorded in Onslow Bay compared to Jacksonville (p = 1). In general, pulse trains recorded at Onslow Bay (mean ± sd: 75.9 ± 13.5 s; 186.9 ± 37.3) and Jacksonville (76.5 ± 10.1 s; 191.3 ± 34.5) were longer and had more pulses per pulse train as compared to pulse trains recorded at Stellwagen Bank (39.9 ± 6.5 s; 85.4 ± 13.6).

## Discussion

### Comparison of ambient noise levels and detection range estimation

Ambient noise levels varied between sites and seasons, with the southernmost recording site experiencing lowest median noise levels during the selected analysis periods (Table 1) and with lower median noise levels in winter as compared to summer and autumn measurements. This spatial gradient of decreasing ambient noise levels from north to south along the US East coast matches a recent in-depth analysis of noise levels from ten different sites along the western North Atlantic coast [61]. Estimated detection ranges based on these measured background noise levels differed accordingly. For example, detection ranges of about 20–30 km, estimated for Jacksonville (site 8, Figure 2), are about 10–20 km greater than estimates for Stellwagen Bank, and detection ranges between seasons differed by 5–10 km (Figure 3). These spatio-temporal differences in ambient noise levels have important implications for behavioural and physiological responses to noise, as well as potential communication ranges for minke whales in their different seasonal habitats [62-65]. In addition, this preliminary analysis highlights that increased ambient noise levels will affect signal-to-noise ratio (SNR) and thus influence detection probability and range in different habitats. Such differences will likely not dramatically change large-scale patterns of seasonal occurrence, which were the focus of this study. However, together with site-specific propagation characteristics, they need to be taken into account when absolute numbers of detections are compared on smaller spatial and temporal scales or used to infer absolute or relative abundance of animals [66,67].

### North Atlantic minke whale migration and winter habitats along the US continental shelf

Minke whale pulse trains were recorded at 11 sites throughout the North Atlantic. It is currently unknown what proportion of the population produces pulse trains and whether there are differences between sexes and/or age-classes in pulse train production. Although it is unclear what proportion of the population is represented by this analysis, a recent study at Stellwagen Bank showed general agreement of visual sighting rates and frequency of acoustic detections [55]. Thus, the minimum assumption is that an increase in acoustic detections represents an increase in vocally active individuals rather than a change in behaviour of the population. However, as mentioned above, propagation characteristics and ambient noise levels need to be considered as well, especially in the absence of visual sightings.

**Figure 7:**
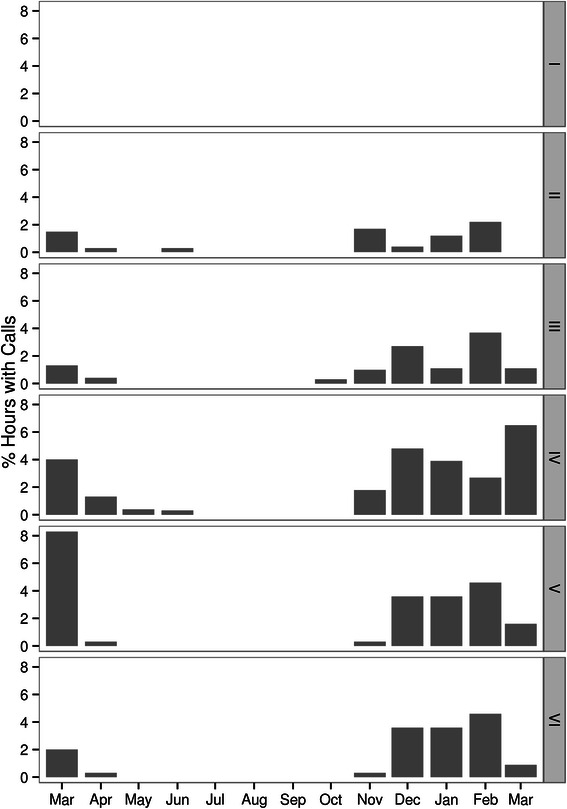
**Minke whale pulse train detections expressed as proportion of hours with detections/month at the Mid-Atlantic ridge.** Panels show different recording sites as labeled in Figure 2. Figure adapted from [49]. Reprinted and adapted with permission of the author.

The results from this study show seasonal variability in minke whale pulse train occurrence along the North American continental shelf consistent with seasonal migratory movement between northern and southern latitudes in summer and winter, respectively. A gradual decrease of detections at sites north of 40° N in late autumn, and an increase in recorded pulse trains in waters between 20° and 30° N during winter and north of 35° N during spring, clearly indicate movement between high-latitude summer feeding grounds and low-latitude winter habitats (Figures 2 and 4). The timing of these movements agree with recent satellite tagging data from Iceland demonstrating the departure of individual minke whales from Icelandic waters from late September to late October [48]. In addition, winter presence in tropical waters and arrival in and departure from these regions closely matched pulse train distribution recorded at the Mid-Atlantic ridge in an earlier study [49] (Figure 7), indicating that minke whales are spread out at low latitudes ranging from the US continental shelf to the Mid-Atlantic ridge during winter. Results from the current study also add further support for the suggested location of a minke whale winter breeding ground offshore the Southeastern US and the Caribbean [41,54]. Recent winter sightings from aerial surveys in the South Atlantic Bight included sightings of mother-calf pairs off North Carolina and Florida. These sightings were corroborated by long-term sighting and stranding records of calves, which occurred primarily during winter and spring in this region [47]. Together, these data confirm the presence of minke whales offshore the Southeastern US shelf break and emphasize the importance of this region as a potential breeding and calving ground for this species. The general seasonal pattern of migration that was observed along the US North Atlantic shelf break can be observed at the Mid-Atlantic ridge as well, with highest detection rates on the southernmost locations (Figures 2 and 7) during winter. Interestingly, no pulse trains were recorded on the northeastern most hydrophone, located at Latitude 32° N. This suggests that minke whales in the western North Atlantic may pass this location further to the west and begin to spread out towards the Caribbean in the west and the Mid-Atlantic ridge to the east, once they have reached lower latitudes.

Acoustic array data from New York (site 5; Figure 2) and Jacksonville (sites 7 & 8; Figure 2) demonstrate that minke whales preferentially migrate in the deeper waters to the east of the continental shelf break (Figure 5). A similar spatial distribution has been found at Stellwagen Bank [55]. Although better sound propagation characteristics in deeper waters could be partly responsible for these observed patterns in detections [68], the general scarcity of winter sightings and results from recent aerial surveys sighting minke whales exclusively offshore of the continental shelf break [47] indicate that differences in seasonal acoustic detections reflect actual animal distribution.

**Figure 8:**
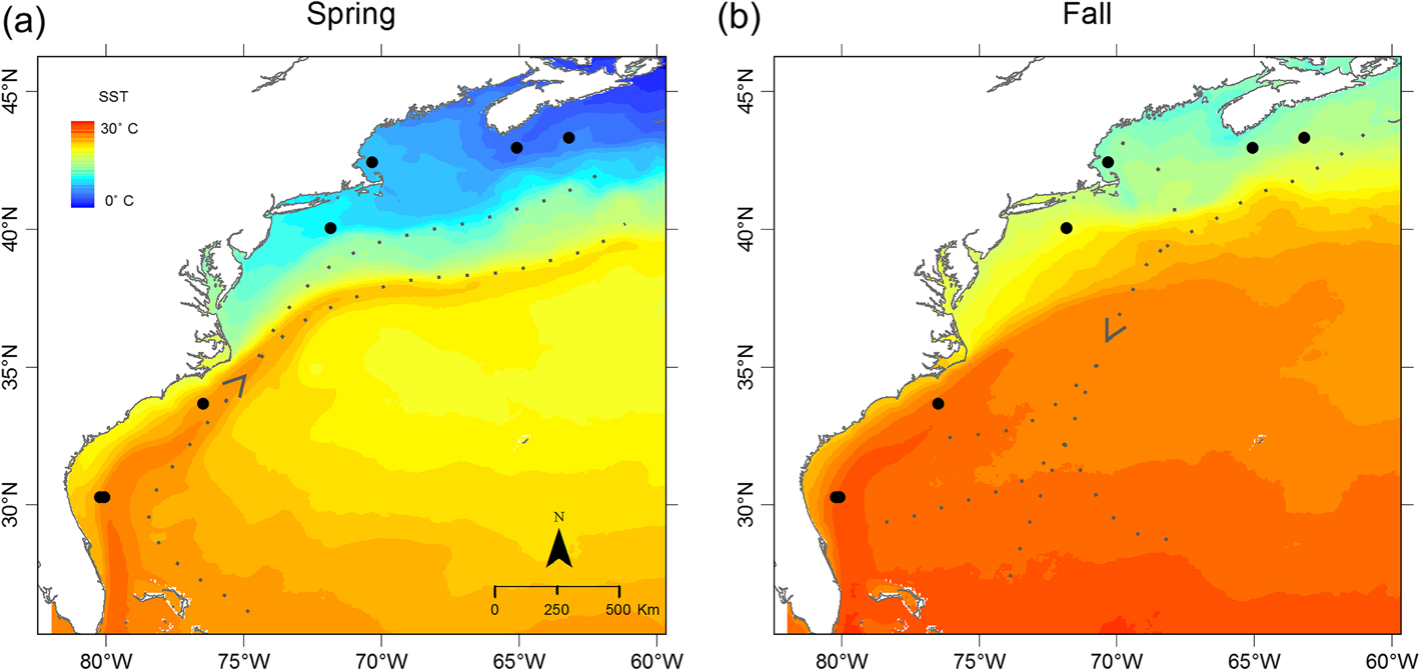
**Maps of Sea Surface Temperature (SST) data for 2012, averaged by season (a) spring (March-May) and (b) autumn (September-November).** Black dots represent recording sites 2–9 as analyzed in this study (see overview map in Figure 2) and dotted lines show hypothetical migration pathways based on frequencies of acoustic detections at different recording sites. For sea surface temperature (SST) raster generation, daily, 1 km resolution, level 4 GHRSST data were downloaded and aggregated into monthly climatological SST rasters using the Marine Geospatial Ecology Tools (MGET) [72]. Monthly SST rasters were then averaged to create seasonal climatological SST rasters. Data available at: http://podaac.jpl.nasa.gov/dataset/JPL_OUROCEAN-L4UHfnd-GLOB-G1SST.

One of the most surprising results of this study was the relative scarcity of detections in the New York (site 5; Figure 2) autumn data compared to a peak in detections during spring in this region. This seasonality is contrary to the one found at Stellwagen Bank (site 4; Figure 2), located about 200 miles further to the north (Figure 4). Yet, similar to seasonal patterns off New York, a peak in late winter and springtime detections compared to less detections during early winter months was observed in Onslow Bay, North Carolina (site 6; Figure 2) (Figure 4). Combined, these data suggest that minke whales are distributed closer to the shelf break edge during their northbound migration in spring than during their southbound migration in autumn. However, high numbers of detections in data from Jacksonville (sites 7 & 8; Figure 2) and several detections at the inshore Saba Island site (site 10; Figure 2) indicate that whales are moving closer inshore again during winter months (Figure 4). Similar observations of a clockwise movement, with minke whales entering southern winter grounds from the northeast and moving in a westerly direction towards the US shelf break, have also been described from IUSS-SOSUS acoustic array data [50]. A possible explanation for these clockwise movements in western North Atlantic wintering grounds is that during spring minke whales are following the northward currents of the Gulf stream, while during autumn, after leaving seasonal feeding habitats north of 40° N, they follow a more directed southerly route, thereby reaching warmer waters more quickly and avoiding swimming against the Gulf Stream that may have surface currents velocities of up to 2.6 m/s [69] (Figure 8). A northward migration following the Gulf Stream and the shelf break could also explain the absence of minke whale pulse train detections and visual observations at Stellwagen Bank (site 4; Figure 2) [70] and at recording sites in Nova Scotia (sites 2 & 3; Figure 2) (Figure 4) during spring, since minke whales may be moving along the shelf break and not spread out into coastal feeding habitats, such as the Gulf of St. Lawrence [71], until they reach higher latitudes.

It has been well documented that minke whale presence is related to prey distribution in their summer foraging grounds, where they feed primarily on pelagic shoaling fish such as sand lance (*Ammodytes sp.*) and herring (*Clupea harengus*) [34,38,71,73]. However, it has also been shown that baleen whales may pause migration and feed on the way to or from their summer habitats [16,74]. Following the Gulf Stream (Figure 8) might also be related to prey availability on their migratory pathway and could have energetic advantages for western North Atlantic minke whales that exploit the main current direction. Similarly, acoustic presence of minke whales off Nova Scotia (sites 2 & 3; Figure 2) and at Stellwagen Bank (site 4; Figure 2) during autumn migration (Figure 4; Figure 2) may be related to prey availability. Although low site fidelity [36] and swimming speeds [75] indicate that both of these areas are part of the migration route, whales might take advantage of herring spawning activity peaking from late August to mid-October in this region [76], while en route to lower latitudes.

The potential relationship between minke whale migration and the Gulf Stream may have important implications in a changing climate. In 2011 warm waters originating in the Gulf Stream were observed much closer to the shelf break south of New England than in previous years [77]. Such shifts in temperature may affect primary productivity, can result in major shifts of fish populations [78,79], and ultimately impact the distribution and abundance of top predators. For example, changes in sea surface temperature (SST) during an El Niño event in the Southern Ocean have been related to reduced calving rates in Southern right whales (*Eubalaena australis*), likely due to reduced prey availability [80]. If minke whales are indeed following the warmer surface waters of the Gulf Stream, a change of its location may potentially shift their migration path and change their overlap with other species, including important prey, as well as anthropogenic activities.

### Absence of pulse trains from summer feeding grounds and the eastern North Atlantic

Very few to no minke whale pulse train detections were recorded during summer in any of the datasets (Figure 4). In traditional summer feeding habitats, such as the Mingan Islands in the Gulf of St. Lawrence [71], only five acoustic detections were made during two months of recording (Figure 4) despite a regular presence of minke whales in the area (Risch D, pers. obs.). In Davis Strait, no detections were made and in Southwest Iceland only one pulse train was detected in the month of October (Figure 2). The absence of acoustic detections from these areas could be related to a switch in behaviour at this time of year and reduced or changed vocalization activity during summer when whales are primarily feeding. For example, in humpback whales, only males produce songs in a reproductive context [81], which, although more prolific on summer feeding grounds than previously thought, shows a strong seasonality, with reduced occurrence during summer when whales are actively feeding [82]. During summer, humpback whale vocal presence on higher latitude feeding grounds is better represented by ‘social sounds’, which are produced by males and females [83,84]. Similar seasonal patterns have been found for fin whale song on high latitude feeding grounds [9]. Therefore, more data on the behavioural function of the full vocal repertoire of minke whales, which in the North Atlantic may include low-frequency downsweeps and other sounds [51,52], is needed to evaluate whether a switch in behaviour may be responsible for the absence of pulse train detections in these areas.

An alternative explanation for the absence of pulse trains in higher latitudes is that the proportion of the population producing pulse trains is not adequately captured in those areas. In the Mingan Islands, Gulf of St. Lawrence (site 1; Figure 2) the sex ratio appears to be heavily skewed towards females [85]. In Davis Strait, to the west of Greenland (site A; Figure 2), sexual segregation results in a higher proportion of females as compared to regions east of Greenland and females are also found in higher latitudes than males [86]. In humpback whales and, both blue and fin whales, only males produce songs that are thought to serve in a reproductive context [87,88]. Although it is currently unknown whether minke whale pulse trains are sex-specific also, the absence of pulse train detections in two different areas with a high proportion of females suggests that they may be.

An absence of minke whale pulse trains from recording sites in the Strait of Gibraltar in the Eastern North Atlantic (sites D + E; Figure 2) may represent an actual absence of minke whales at these sites. Although minke whales have been observed to enter the Mediterranean Sea [89], sightings are generally few, and it is unclear whether minke whales have a year-round presence or enter the Mediterranean Sea seasonally [39]. However, only three months of winter recordings were available for these sites (Table 2) and for final conclusions, recordings at other times of year need to be explored, since migrating whales might have been missed by the restricted sampling duration.

**Table 2:** **Summary of recording sites, geographic locations, depth, available recording days, duty cycle (recording period/time period), sample rate and recorder type**

**Site**	**Location**	**Depth (m)**	**Recording period (n days)**	**Duty cycle (min)**	**Sample rate (kHz)**	**Recorder type**
*Davis Strait (A)*	*67.24/-58.8*	*350*	*10/23/06–10/05/07 (348)*	*Cont.*	*2*	*HARU* ^*1*^
SW Iceland (B)	58.0/-26.0	800	05/16/07–07/25/08 (437)	Cont.	2	HARU^1^
Gulf of St. Lawrence (1)	50.25/-64.22	25	06/23/10–09/13/10 (83)	Cont.	2	MARU^2^
Roseway Basin (2)	42.97/-65.06	145	07/02/04–08/17/05 (412)	Cont.	2	HARU^1^
Emerald Basin (3)	43.34/-63.16	153	07/02/04–10/13/05 (469)	Cont.	2	HARU^1^
Stellwagen Bank (4)	42.45/-70.31	71	12/18/08–03/19/10 (457)	Cont.	2	MARU^2^
New York (5)	40.05/-71.82	90	02/29–05/15/08; 08/29–03/05/09 (266)	Cont.	2	MARU^2^
*Azores (C)*	*38.54/-29.04*	*190*	*04/10–09/17/10; 09/29/10–5/19/11(424)*	*1.5/30*	*50*	*EAR* ^*3*^
*Cape Espartel East (D)*	*35.87/-6.20*	*340*	*10/28/08–01/30/09 (95)*	*5/10*	*2*	*EAR* ^*3*^
*Strait of Gibraltar West (E)*	*36.03/-5.42*	*100*	*10/28/08–01/30/09 (95)*	*5/10*	*2*	*EAR* ^*3*^
Onslow Bay (6)	33.68/-76.48	335	04/24–08/09/09; 11/08/09–04/20/10 (271)	5/10	200	HARP^4^
*Savannah (F)*	*31.83/-80.70*	*17*	*11/18/09–03/16/10 (119)*	*Cont.*	*2*	*MARU* ^*2*^
Jacksonville 1 (7)	30.27/-80.06	91	02/22–07/30/10; 08/26/10–01/25/11 (312)	5/10	200	HARP^4^
Jacksonville 2 (8)	30.28/-80.06	305	09/13–10/08/09; 12/03/09–01/07/10 (62)	Cont.	2	MARU^2^
Jacksonville 3 (9)	30.34/-81.21	17	11/19/09–06/04/10 (197)	Cont.	2	MARU^2^
Saba Bank (10)	17.51/-63.19	30	10/27/11–04/28/12 (185)	30/120	16	MARU^2^

Sites at which no minke whale pulse train detections were made are in italics. See the following references for details about recorder electronics and sensitivities: (1) HARU phones: [90]; (2) Marine Autonomous Recording Unit (MARU): [91]; (3) Ecological Acoustic Recorder (EAR): [92]; (4) High-frequency Acoustic Recording Package (HARP) [93].

Very little is known about minke whale migration in the middle and eastern North Atlantic, but it has been suggested here too, that migration takes place in open, offshore waters [39,46] and recent satellite tracking data are in support of this idea [48]. The absence of minke whale pulse trains from recording sites located in the Azores, where minke whales are occasionally sighted during spring and early summer [16], is thus surprising. However, most minke whales may be passing the archipelago too far offshore to be acoustically detected. In contrast, from November to June, minke whale pulse trains were frequently recorded at recorders deployed to the east and west of the Mid-Atlantic ridge [49] (Figure 7), indicating that minke whale breeding grounds extend eastwards from the Caribbean to at least the Mid-Atlantic ridge.

Finally, the absence of minke whale pulse trains from recording sites in the eastern North Atlantic may be in part related to geographic differences in vocalizations that could not be resolved by the automated detector used in this study. For example, while [53] found mainly speed-up pulse trains in data from the Caribbean, data from Stellwagen Bank showed a predominance of slow-down pulse trains [55]. Although the automated pulse train detector used here was built on data originating from Stellwagen Bank, North Carolina and Jacksonville, most pulse trains used for training were of the slow-down type. There are differences in the frequency distribution between these two types [53], thus a concentration on slow-down pulse trains from the western North Atlantic for detector development might have influenced detector performance at other sites, especially those that are geographically more distant.

### Geographic variation in signal structure

Although a thorough comparison of the full vocal repertoire was beyond the scope of this study, preliminary data from Onslow Bay, North Carolina suggest that the main minke whale call categories found at Stellwagen Bank [55] are present at southern recording sites also [94]. A comparison of pulse train type sd3 recorded at Stellwagen Bank, North Carolina and Jacksonville (Figure 1) shows evidence for geographic variability in signal structure with pulse trains being about 30 seconds longer and containing about 100 more pulses on southern recording sites as compared to recording sites in higher latitudes (Figure 6). Although pulse train duration measurements for the Jacksonville site were correlated with SNR (Additional file 1: Figure S1), and are thus a minimum estimate, these results corroborate pulse train measurements from the Caribbean which were similar in length and number of pulses to pulse trains from North Carolina and Jacksonville [53]. As mentioned above, the majority of pulse trains found in the Caribbean were of the speed-up type as opposed to a majority of slow-down types in northern sites. The reasons for this difference are unclear but individual differences in call type production could be partly responsible [75]. A more in depth comparison of minke whale vocal repertoire and call type occurrence may help to elucidate more differences and similarities between sites and shed light on the behavioural function of these sounds. Although it is unclear whether the significant increase in signal duration is true for all types of pulse trains, none of the pulse trains from Stellwagen Bank measured during an earlier study [55] lasted as long as some of the pulse trains recorded on southern recording sites in this study.

Testosterone mediated male singing behaviour, increased signal duration and complexity are well documented in a range of vertebrates [95-97]. The increased duration of minke whale pulse trains on potential winter breeding grounds, the general scarcity of these signals on feeding grounds and their increased occurrence during autumn migration, when testosterone levels in adult males are rising [98], are all strong indicators for a reproductive function of these sounds. As argued above, there is also some evidence indicating that females are not producing these sounds. However, more data from breeding grounds, higher latitude feeding grounds with an even distribution of sexes or acoustic tag recordings from individuals of known sex are necessary to conclusively answer the question of sex-specificity and behavioural context of minke whale pulse trains.

## Conclusion

This study confirms the seasonal migration of North Atlantic minke whales offshore the eastern US continental shelf in spring and autumn and their winter presence in southeastern US and Caribbean waters. The identification of a potential breeding ground offshore of the southeastern US may enable more directed genetic sampling of this species in order to help elucidate population structure [43], with potentially important implications for current management of this species in the North Atlantic Ocean. Another important result of this study is the scarcity of pulse train detections north of 50° N during summer, when minke whales are abundant in coastal feeding habitats. These results either indicate a switch of vocal behaviour at this time of year, or, if signals are sex-specific, illustrates the sexual segregation of North Atlanic minke whales on their feeding grounds as described in earlier studies [86].

These results emphasize the feasibility of using passive acoustic monitoring (PAM) networks for investigating the spatial and seasonal distribution of pelagic baleen whale species that are difficult to survey by visual methods alone. However, in order to interpret these detection results beyond presence/absence of species and in the context of animal population density, there is a clear need for extended baseline data collection. Currently missing data include vocalization rates based on group size, in different behavioural contexts, by sex and age class, as well as data collected at different seasonal and spatial scales [99]. These data are extremely scarce for most marine mammal species. Yet, recent developments in technologies such as digital recording tags [88,100-102], as well as analysis techniques for localization and tracking of individual animals using passive acoustic data [103-105] may help to close some of these current data gaps in the future.

## Methods

### Acoustic data collection

Long-term acoustic data for this project were collected across multiple years and at 16 different sites throughout the North Atlantic Ocean using a variety of different recording packages (Figure 2, Table 2). Data availability and temporal consistency was limited by the goals of the various long-term monitoring projects, with differing analysis targets, which contributed data to this large-scale meta-analysis [11,55,58,106-109]. However, the main objective of this project was to explore large-scale migration and characterize the seasonal occurrence of minke whale pulse trains at different sites throughout the North Atlantic. Thus, recording periods were selected to maximize the overall spatial coverage and the seasonal coverage within each site, rather than to keep annual consistency. Table 2 summarizes recording locations, available recording days, recording schedules, sample rates and equipment types used. Most recording effort was concentrated along the United States (US) east coast and used four types of bottom-mounted recorders deployed in depths ranging from 17 to 800 meters (Figure 2). While most recorders sampled continuously at 2 kHz, some recordings were scheduled to record every 1.5 to 30 minutes and sampling rates ranged up to 200 kHz for some recorders (Table 2). All data were downsampled to 2 kHz before automatic detection and further data processing.

### Data analysis

#### Automatic detection

North Atlantic minke whales are known to produce up to seven types of low-frequency pulse trains, which can be assigned to three major categories (slow-down, constant and speed-up pulse train), based on varying interpulse interval structure (IPI) [53,55]. An automated detector was developed to examine selected recordings for the presence of these pulse trains. The automatic detection consisted of a multi-stage process based on spectrogram intensity binarization, energy projection, feature extraction and classification [110]. While the detection stage was designed for general pulse train detection, a Rippledown Rule (RIDOR) learner [111] was trained to identify minke whale pulse trains, taking into account, but not distinguishing among, the different types of pulse trains. A total of 18 basic features were extracted from each detected event and passed to the RIDOR for classification (see details in [110]). The overall false negative rate (FNR) of the detector was assessed in an earlier study and was found to be 27% (647 out of 2428 true positive (TP) detections), with 181 false positive (FP) detections in 120 hours (or 29,847 signal slices) of evaluated data [55]. Experienced data analysts (GD & DR) manually verified all detected pulse trains using the MATLAB (Mathworks, Natick, MA) based custom software program SEDNA [112]. All false positive detections were removed from each analyzed dataset.

#### Ambient noise levels and estimated maximum detection ranges

Since variations in ambient noise levels (NL) by site and season can have a profound impact on the detection probability of acoustic signals [67], an exploratory ambient noise analysis was conducted for three recording sites (sites 4, 5, 8: Stellwagen Bank, New York, Jacksonville 2; Figure 2), for which equipment calibration information was available. LTSpec, a custom-written MATLAB script [113], was used to aggregate and compute long-term spectrograms and extract absolute root-mean-square (RMS) received levels over a frequency band encompassing six third-octave bands (center frequencies at 100, 125, 160, 200, 250, and 315 Hz). This frequency band was chosen to include most energy content of minke whale pulse trains, which is concentrated between 50 and 300 Hz (Figure 1) [53,55]. Site-specific and seasonal week-long data (Additional file 2: Table S1) were aggregated over a time period of ΔT = 600 s. Spectrograms were created using a sampling rate of 2000 Hz, a FFT size of 2048, and a Hanning window function, resulting in a frequency resolution of 0.98 Hz. Ambient noise levels (NL) were used to estimate maximum detection ranges of minke whale pulse trains. Assuming source and receiver depths of 20 m, an average source level of 165.4 dB [75] and pulse length of 0.1 s, signal propagation was modeled for an omni-directional source of 120 Hz over 8 horizontal radii and for all four seasons, using a BELLHOP acoustic simulation model implemented in ESME [60], and environmental databases provided by the Oceanographic and Atmospheric Master Library (OAML) (available at http://esme.bu.edu/). The maximum propagation radius was selected and compared to all measured ambient noise levels. The maximum detection range was then estimated as the point at which SNR (RL-NL) equals zero and ranges for different sites and seasons were compared using empirical cumulative distribution functions, calculated with function ecdf of the R v. 3.1 stats package (available at www.R-project.org).

#### Geographic variation in acoustic features

A subset of non-overlapping detections of high signal-to-noise ratio [SNR >10 dB] from three sites (n = 68, 26, 35 for sites 4, 6, 8: Stellwagen Bank, Onslow Bay, Jacksonville 2; Figure 2) were selected to measure and compare slow-down pulse train type sd3 as defined by [55]. This type of pulse train is characterized by a bimodal distribution in IPI, peaking at 0.4 and 0.7 s (Figure 1) [55]. It was selected for this geographic comparison, since it was one of the most frequently occurring and easily distinguishable pulse train types in all datasets [55]. Acoustic data for this analysis were bandpass filtered from 30 to 800 Hz to remove environmental noise and signals from other species. Spectrograms (FFT size: 512 points, 96.9% overlap, Hanning window, time resolution: 8 ms, frequency resolution: 4 Hz) were created and analyzed using Avisoft-SASLab Pro 5.1 (Avisoft Bioacoustics). The automatic parameter measurement tool was used to measure pulse train duration and identify the total number of pulses per pulse train using an amplitude threshold of −30 to −55 dB sound pressure level (SPL) relative to the maximum SPL in the sound file. The threshold was manually adjusted to ensure the detection of most pulses within a pulse train. Given that the data were not normally distributed (Saphiro-Wilk test), the hypothesis that mean pulse duration and number of pulses differed between sites was tested using a Kruskal-Wallis test. Wilcoxon rank-sum tests with Bonferroni corrections for multiple testing were used for post-hoc comparisons between pairs of sites. All statistical analyses were conducted using R v. 3.1. In order to select high quality signals for this analysis and test whether SNR affected the duration measurements, SNR of the whole signal was measured within a selection box including the signal and time periods just before and after a pulse train, using the MATLAB based sound analysis tool Osprey [114].

#### Seasonal and spatial variation

One recorder per site and deployment period was selected in order to examine seasonal patterns of minke whale pulse train occurrence. Since preliminary data from migration and winter habitats suggest an offshore distribution of minke whales [47,55], at sites where multiple recorders were available, preference was given to the recorders deployed farthest from shore. All data from sites with at least five detections were binned and plotted by day. In addition, the seasonal and geographic patterns of pulse train occurrence along the US east coast continental shelf, where most recording effort was concentrated, were compared to seasonal minke whale pulse train occurrence from the Mid-Atlantic ridge [49]. In order to simplify the description of seasonal patterns of pulse train occurrence the four seasons will be defined as follows for the remainder of the paper: winter = December to February, spring = March to May, summer = June to August and autumn = September to November.

For the New York recording site (site 5; Figure 2), data from nine recorders, stretching from west to east across the continental shelf, were available for analysis. For Jacksonville, data from four recording units, deployed from west to east, were available (sites 7–9; Figure 2). For these two geographic sites, the total number of detections was evaluated for all available recording units in order to characterize the spatial distribution of minke whale pulse train detections as a function of distance from shore and shelf break.

## Additional files

Additional file 1: Figure S1. Scatterplots and regression lines (CI = 95%) of Signal-to-Noise Ratio (SNR) in dB against pulse train duration and number of pulses/pulse train, comparing data from Stellwagen Bank (SBNMS), Massachusetts (site 4); Onslow Bay, North Carolina (site 6); and Jacksonville, Florida (site 8). Additional file 2: Table S1. Overview of weeks analyzed for ambient noise analysis.
