# Supplementary figures and images for: Seasonal migrations of North Atlantic minke whales: novel insights from large-scale passive acoustic monitoring networks
Source: Mov Ecol. 2014 Nov 18;2:24. doi: 10.1186/s40462-014-0024-3 (PMC4337769; doi:10.1186/s40462-014-0024-3)

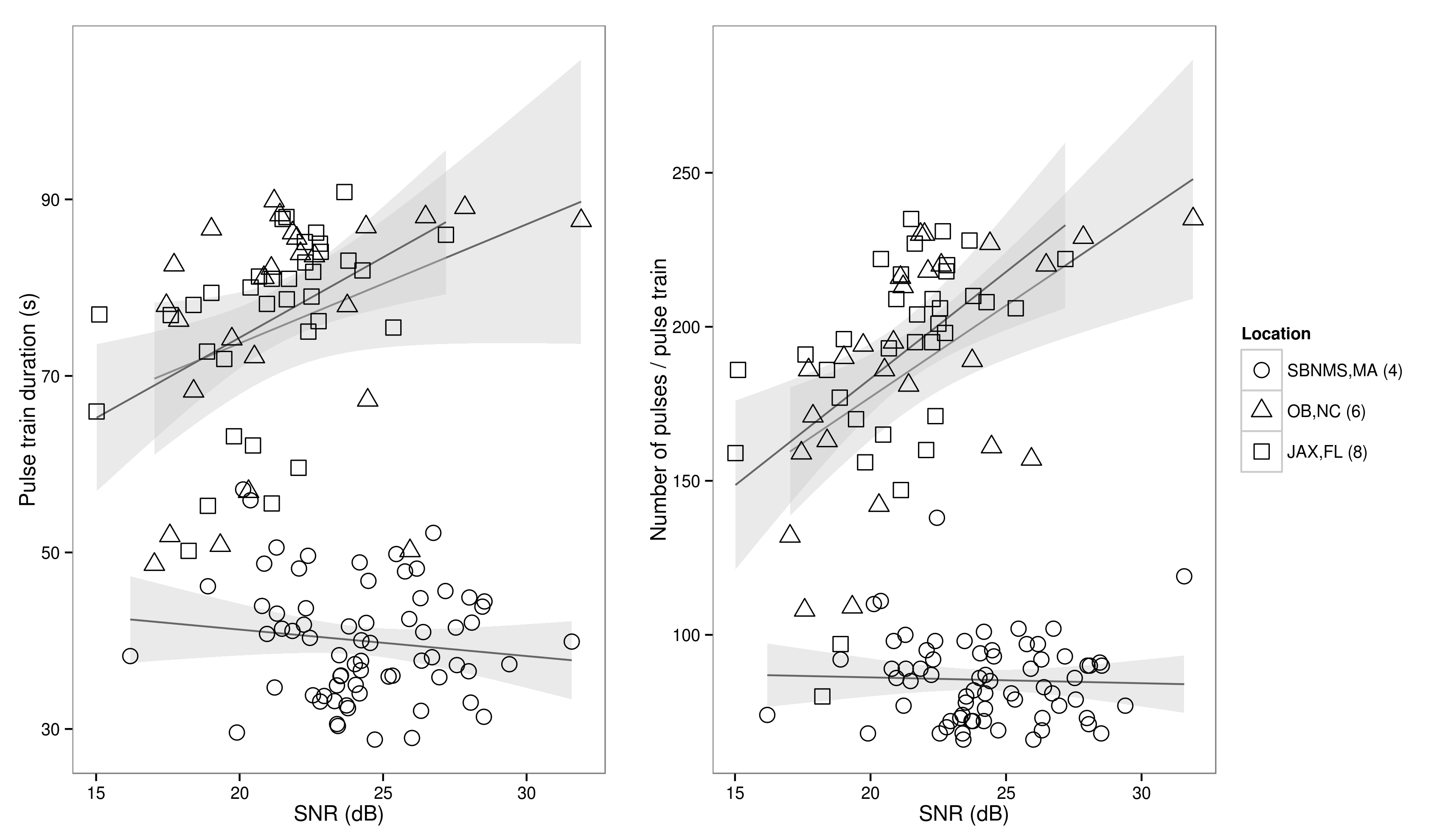

Supplement: Additional file 1: Figure S1. — Scatterplots and regression lines (CI = 95%) of Signal-to-Noise Ratio (SNR) in dB against pulse train duration and number of pulses/pulse train, comparing data from Stellwagen Bank (SBNMS), Massachusetts (site 4); Onslow Bay, North Carolina (site 6); and Jacksonville, Florida (site 8). [file 40462_2014_24_MOESM1_ESM.tiff]
