# Supplementary material for: Seasonal migrations of North Atlantic minke whales: novel insights from large-scale passive acoustic monitoring networks
Source: Mov Ecol. 2014 Nov 18;2:24. doi: 10.1186/s40462-014-0024-3 (PMC4337769; doi:10.1186/s40462-014-0024-3)
Supplement: Additional file 2: Table S1. — Overview of weeks analyzed for ambient noise analysis. [file 40462_2014_24_MOESM2_ESM.doc]

| **Location (Site)** | Winter | Spring | **Summer** | **Autumn** |
| --- | --- | --- | --- | --- |
| SBNMS (4) | 01/01/09-01/07/09 | 04/01/09-04/07/09 | 08/01/09-08/07/09 | 11/01/09-11/07/09 |
| NY (5) | 01/01/09-01/07/09 | 04/01/08-04/07/08 | 08/30/08-09/05/08 | 11/01/08-11/07/08 |
| JAX (8) | 12/18/09-12/24/09 | -- | -- | 10/01/09-10/07/09 |

**Table S1.** Overview of weeks analyzed for ambient noise analysis.
